# Comparing CDC's surveillance definitions and CPIS score in diagnosing ventilator-associated pneumonia: an observational study

**DOI:** 10.1186/cc14699

**Published:** 2015-09-28

**Authors:** Álvaro Koenig, Dimitri S Possamai, Fernanda P de Aguiar, Glauco A Westphal, Kenia Fujiwara, Lucas R Ramos, Milton C Filho, Miriam Cristine Machado, Renata Waltrick, Valmir João de S Filho

**Affiliations:** 1 Hospital Municipal São José e Centro Hospitalar Unimed, Joinville, SP, Brazil

## Introduction

The National Healthcare Safety Network/Center for Disease Control and Prevention (NHSN/CDC) published in 2013 a new surveillance protocol in order to standardize the ventilator-associated pneumonia (VAP) confirmation criteria and, consequently, to increase the reliability of indicators in different institutions.

## Objective

To evaluate the degree of agreement of CDC's new surveillance definitions and clinical criteria, using the CPIS score, in diagnosing VAP.

## Methods

From August 2013 to June 2014 all patients on mechanical ventilation for longer than 48 hours in two critical care units in a public and a private general hospital were included in the study. On a daily basis, ventilated patients were evaluated by respiratory physiotherapists using the CPIS score and, independently, by the infection preventionist nurse using CDC's new surveillance definitions. CPIS score = 7 was considered a clinical diagnosis, and, when associated with a semiquantitative culture with 10^4 ^colony-forming units, as a definitive diagnosis of VAP.

## Results

Eight hundred and one patients were admitted to both ICUs during the study period. One hundred and sixty-eight were on mechanical ventilation for more than 48 hours. Thirty-eight patients were diagnosed with pneumonia using the clinical criteria (13.8/1000 patients/day on ventilation). Eighteen of these patients were diagnosed with infectious conditions associated with mechanical ventilation (IVAC) and 14 of them had a diagnosis of probable VAP (5.23/1000 patients/day on ventilation). Compared with clinical criteria, the CDC's surveillance definitions had sensitivity = 0.37, specificity = 1.0, positive predictive value = 1.0 and negative predictive value = 0.84.

## Conclusion

Compared with clinical criteria, using the CPIS score, the CDC's new surveillance definitions had a low sensitivity and may not be appropriate as a surveillance method.

